# A health economic analysis of the management of open lower limb fractures in the elderly

**DOI:** 10.1007/s00590-020-02713-3

**Published:** 2020-06-09

**Authors:** Caitlin Pley, Katie Purohit, Matija Krkovic, Ali Abdulkarim

**Affiliations:** 1 School of Clinical Medicine, University of Cambridge, Cambridge, UK; 2 Addenbrooke′s Hospital, Hills Road, Cambridge, UK

**Keywords:** Trauma, Open fracture, Health economics, Older age, Health systems

## Abstract

**Aim:**

The aim of this study was to investigate the financial implications of the inpatient management of open lower limb fractures in adults over 65 years old. Further, the study compares the calculated cost to the income received by the hospital for these patients and to the existing body of literature.

**Methods:**

This study employed direct inpatient costing analysis to estimate the cost of treating the open lower limb fractures incurred by 58 patients over the age of 65 years treated in our centre (Addenbrooke's Hospital, Cambridge University Hospitals NHS Trust) between March 2014 and March 2019.

**Results:**

The median cost of inpatient care calculated in this study was £20,398 per patient, resulting in a financial loss to the hospital of £5113 per patient. When the results were disaggregated by sex, the median cost for an open lower limb fracture in a male patient was £20,886 compared to £19,304 in a female patient. Data were also disaggregated by the site of injury, which produced a median cost for an open femur fracture of £23,949, and £24,549 and £15,362 for open tibia and ankle fractures, respectively.

**Conclusion:**

This study provides a valuable estimate of the expense of treating open lower limb fractures in patients over the age of 65 years in a Major Trauma Centre in England. The study highlights the large losses incurred by hospitals in treating these cases, and supports revision of the remuneration structures in the National Health Service to adequately cover their cost.

## Introduction

The cost of modern health care is a major public policy issue in many countries worldwide; one that incites widespread public debate and often finds itself centre stage in political campaigns and election manifestos. In the United Kingdom (UK), health care expenditure has more than doubled in the last 50 years [1]. However, more recently, spending on health care has fallen from 9.8% of GDP in 2013 to 9.6% in 2017 [1, 2]. UK government health care spending per capita is near the median for the Organisation for Economic Cooperation and Development (OECD) but is the second lowest of the G7 countries [3]. In the last ten years (2009/10 to 2018/19), National Health Service (NHS) budgets have increased by an average of 1.5% annually, lower than the rate of inflation [4].

In the NHS, hospitals are aggregated in organisational units called Trusts, which are constructed both by geographical area and the specialist functions of the facilities. NHS Trusts are semi-autonomous public sector corporations with independent financial management. The expenditures of the Trust are reimbursed by the central NHS budget based not on the actual expenditures, but on Healthcare Resource Group (HRG) cost codes. HRG tariffs are designed to incentivise providers to reduce their unit costs by finding ways to improve efficiency [5].

The architecture of trauma care in the NHS was restructured in 2012, resulting in the regional centralisation of trauma care into Major Trauma Centres (MTC). MTCs are specialist units designed to provide advanced trauma care, with the necessary facilities and experienced staff to handle major trauma, and are usually based in larger hospitals in a central location within a region. Addenbrooke's Hospital, where this study was conducted, is the MTC of the East of England Trauma Network.

Open lower limb fractures are complex injuries that demand multi-disciplinary specialist care from pre-hospital to community rehabilitation. Patients with suspected open lower limb fractures in the pre-hospital environment are transported directly to the nearest MTC or specialist centre with orthoplastic care, unless the patient's medical state necessitates transport to the nearest emergency department for stabilisation prior to transfer to an MTC. In the hospital setting, initial management comprises an ABCDE assessment, pharmacological pain relief, continuous assessment of vascular status to detect evolving compartment syndrome, irrigation, cover and administration of prophylactic intravenous antibiotics. Surgical treatment consists of debridement, fixation and definitive soft tissue cover of the open fracture. Patients with these injuries generally have long hospital stays and require intensive, long-term rehabilitation and physiotherapy after discharge. The management of these injuries is advised by NICE and BOAST 4 guidelines [6, 7].

There exists a scarcity of literature on the economic facets of trauma management. However, one previous study used direct inpatient costing and patient-level costing approaches to estimate the cost of these injuries in an all-age patient cohort, and calculated total costs of £15,725 and £19,189, respectively [8]. This study also found that there existed a significant mismatch between the reimbursement received by the Trust for the care of these patients and the estimated cost. Another study conducted in the UK in 2011 investigated the cost of free flaps for open tibia fractures and found that the mean cost of a free flap for this type of injury was £12,792, more than double the income received through HRG tariffs [9]. There is no official figure for the cost of inpatient treatment of open lower limb fractures in the UK.

However, health economic and market considerations are increasingly being included in trials for new treatments for orthopaedic injuries. The BESTT (BMP-2 Evaluation in Surgery for Tibial Trauma) trial investigated the applicability of a recombinant form of human bone morphogenetic protein-2 (BMP-2) in the management of open tibia fractures, demonstrating safety and superior efficacy to the standard of care [10]. A further study conducted in the UK, Germany and France subsequently determined that the NHS could save up to €9.6 million per year by using rhBMP-2 in the treatment of Gustilo-Anderson grade III open tibia fractures, considering that the use of rhBMP-2 reduced the cost of treatment from €44,757 to €36,847 per patient in the trial [11].

The NHS operates under immense financial pressure, given the limited fiscal space available for health spending, and should perform under the highest standards of allocative and technical efficiency to be able to maximise the provision of quality patient care. Making evidence-based decisions to enhance cost-effectiveness in the NHS requires the availability of high-quality evidence on the cost of different interventions and subsequent dissemination of this information to front-line clinical decision-makers.

As we are unaware of a previous study for the cost of open lower leg fractures in the specific patient groups that are disproportionally affected, we aim to fill a gap in the literature with this study. The aim of this study is to appraise the cost of the inpatient management of open lower limb fractures in adults over 65 years old, comparing this cost to the income received by the hospital for these patients and to the one piece of existing literature found on the cost of treating these injuries in a patient cohort with mean age of 40 years [8].

## Methods

### Patient cohort

Fifty-eight patients over the age of 65 years were identified through the England Trauma Area Network (TARN) database. All were treated for open lower limb fractures at Addenbrooke's Hospital, a tertiary referral hospital in Cambridge (UK), between March 2014 and March 2019. The patient data including date of birth, date of surgery, mechanism of injury, diagnosis and details of the inpatient stay and treatment were extracted from the hospital's electronic record system Epic.

### Cost calculation

The costing method used for this study was direct inpatient costing, as part of a cost-benefit analysis investigating the impact of the cost of treating open lower limb fractures in older patients on hospital revenue [12]. Direct inpatient costing uses data from individual patients, collected retrospectively, to calculate the price of a number of components of care. The components used in the direct inpatient costing calculation in this study were pre-defined and constant across all patients. They were selected to represent the bulk of the total inpatient cost and are generally items that vary for each patient. Since direct inpatient costing includes only a limited number of components of the total inpatient costs, the method unavoidably produces an underestimate.

There are two alternative costing approaches that could also be employed to produce a cost estimate for a patient cohort. Patient-level costing is similar to direct inpatient costing, but more comprehensive and as such its use is limited by the availability of data in the hospital's clinical information system, as it follows an individual patient's journey throughout their time in hospital and takes into account more individualised aspects of care that are not constant across all patients in the cohort. Studies that used both direct inpatient costing and patient-level costing approaches found that patient-level costing yielded higher costs, by several thousand GBP per patient, than the figures obtained with direct inpatient costing [8, 13]. A third approach to costing inpatient care, which was not utilised in this study, is service line costing. Service line costing is a less time-intensive approach that considers the cost of patient care at the aggregate level of a particular hospital division or department, comparing the division's incomes and expenditures to determine the cost of providing patient care. This approach would be inaccurate because modern patient care is multi-disciplinary, meaning that it does not occur in silos of divisions or departments, and thus the expenditures of just one division would not account for the full cost of treating a patient.

Direct inpatient costing, as employed in this study, was composed of the following components of patient care:

#### Inpatient stay

The cost of inpatient stay was calculated by the number of nights spent on the orthopaedic ward (£169/night) and the critical care ward (£899/night) per patient, using inflation-adjusted bed-night figures from the NHS Trust (Cambridge University Hospitals) where care was delivered.

#### Theatre time

The length of all surgical interventions was recorded in the patient case notes on Epic at the time of the interventions. To calculate the cost of using the operating theatre, the rate of £18.98/minute was used, a figure developed by the Scottish Surgical Network in 2011 [14]. This figure has also been used in a number of other recent studies performing cost analyses of surgical care [8, 15–17], although it is likely to be an underestimate [14]. Calculating a more up-to-date figure would be a useful exercise, as small changes in the cost per minute would have a large impact on the overall operative costs since these injuries often require multiple lengthy operations. However, this undertaking is beyond the scope of this paper.

#### Surgical consumables and implants

The cost of all surgical consumables and implants used in the surgical treatment of the open fractures was calculated separately for each case, using the patient's record which detailed the type and supplier of each consumable and implant used. The prices for these items were obtained directly from theatre procurement, and therefore reflect the exact price paid by the Trust. Negative pressure wound therapy devices were only used in a limited number of cases with gross wound contamination. Addenbrooke's Hospital, being a major trauma centre, provides tertiary-level orthoplastic care and the combined approach to the care of these patients is to achieve definitive skeletal and soft tissue reconstruction in a single stage and as soon as possible, as recommended by the British Orthopaedic Association and the British Association of Plastic, Reconstructive & Aesthetic Surgeons [18]. In the few cases where negative pressure devices were used, these have been included in the cost of care.

#### Theatre kits

The cost of non-disposable theatre equipment, including replacement and sterilisation costs, could not be calculated at the individual level of each patient because the theatre kit used in the surgical care of these patients is sterilised and reused between cases. Therefore, the contribution of this component to the final direct inpatient cost is calculated as a mean per patient using the aggregate cost at the department level. Theatre kits are capital investments, often purchased many years ago, with limited replacement costs. In the 2019/2020 financial year, the trauma and orthopaedic surgery department spent £760,027 on new theatre equipment. The cost of sterilisation comprises up-front and long-term operating costs, including electricity, water, cleaning solutions and human resources. The Trust spent £3.6 million on sterilisation in the 2019/2020 financial year, of which trauma and orthopaedic surgery accounted for £718,000. The trauma and orthopaedic surgery department of the Trust treated 4781 patients in this same year.

#### Physiotherapy

The cost of inpatient physiotherapy was calculated using average hourly rates (£53/hour), as obtained from the Unit Costs of Health and Social Care [19].

The direct inpatient costing methodology used in this study, therefore, does not include medications, imaging, additional personnel outside of the core team and the overhead costs of running a hospital. It also does not include treatment received at other centres, which is often the case with these complex polytrauma patients due to the regional architecture of trauma care in the United Kingdom.

### Analysis

The cost of treating open lower limb fractures in older adults (65 years and older) was evaluated as a total patient cohort and median and mean figures were obtained for the cohort. The obtained median cost was also compared to the existing literature. The patient cohort of this study was also analysed according to sex (male, female) and site of fracture (femur, tibia or ankle). Group medians and means were used to compare patient sub-groups. The distribution of the data was wide, and apart from two outliers, approximately normally distributed. The two outliers were patients who had significantly higher costs of care, at £67,078 and £72,665 respectively, and it was determined that these two outliers should remain included in the study because open lower limb fractures are complex injuries that in a minority of patients require costly revision surgeries. These outliers are the reason why the mean (£22,373) of the total patient cohort is higher than the median (£20,398). Without the outliers, the range of costs was £3338 to £45,640 and approximately normally distributed, with a mean of £20,351, very similar to the median of the total cohort. For these reasons, this study presents both the mean and median in the presentation of its results, and uses the median to calculate the difference between cost and income, as it represents a more accurate reflection of the cost difference incurred for a 'typical' patient with this type of injury in our centre. This approach is also consistent with the existing literature, and aids in the comparison of the findings of this study to previously published figures which also used the median to compare cost and income. A previous study has calculated the average reimbursement for an open lower limb fracture in the NHS using the latest Healthcare Resource Groups (HRG) codes [8]. The average margin per patient was calculated by subtracting the median cost derived from Direct Inpatient Costing from the average income for each case.

## Results

### Patient demographics

**Fig. 1:**
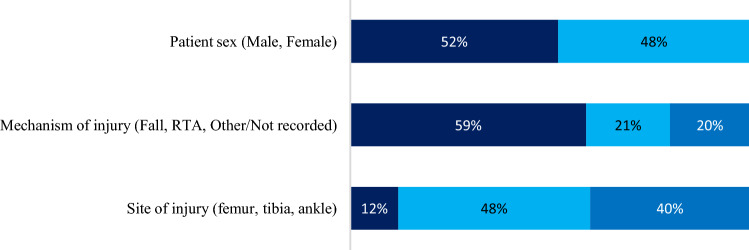
Patient demographics. Visualisation of patient demographics showing the percentage of patients in each demographic category

The mean age of the patient cohort was 72.7 years (range 65–94 years). 30 (52%) patients were female and 28 (48%) patients were male. The most common mechanism of injury was a fall (34 patients, 59%), followed by a road traffic accident (12 patients, 21%). The mechanism of injury was not recorded in 10 patients. Our findings are consistent with a recent study, which found that 62.5% of major trauma in older persons (65 years and older) is caused by a low fall [20]. The sites of fracture were the femur in 7 patients (12%), tibia (condyles and shaft) in 28 patients (48%) and the ankle (medial and/or lateral malleolus, talus) in 23 patients (40%) (Fig. 1).

### Direct inpatient costs

The total cost of treating this patient cohort (58 patients) was £1,297,633. The total income to the hospital for the patient cohort, based on an average income for this injury type, was £886,530, resulting in a total loss to the hospital of £411,103.

The median cost of treating an open lower limb fracture in this cohort was £20,398 per patient. The mean cost was £22,373 per patient. Using the median cost, the average loss to the hospital per patient was £5113.

When the data were disaggregated by sex, the median cost for an open lower limb fracture in a male patient was £20,886 (mean £23,303) compared to £19,304 (mean £21,550) in a female patient (*p* = 0.34). Data were also disaggregated by fracture site, where the median cost of an open femur fracture was £23,949 (mean £23,119), the median cost of an open tibia fracture was £24,549 (mean £28,990) and the median cost of an open ankle fracture was £15,362 (mean £14,862).

### Cost breakdown

**Fig. 2:**
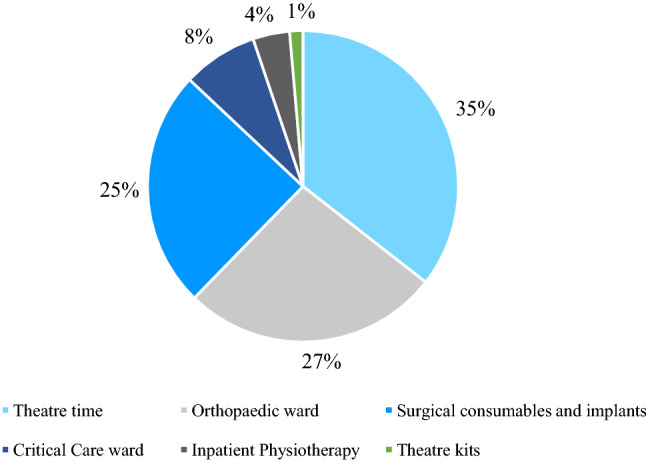
Cost breakdown by direct inpatient costing component. Percentages indicate the share of the total cost per patient accounted for by each of the components used in the analysis

The cost of treating an open lower limb fracture in our centre was also broken down by component. Table 1 shows the relative contributions of the different components of the costing analysis to the final average (mean) cost, sorted by relative contribution of the component. The highest cost to the hospital was theatre time (£7952). Inpatient stay on the orthopaedic and critical care wards cost £5985 (35.47 nights) and £1743 (1.94 nights), respectively, on average (mean) per patient. The mean cost of surgical consumables and implants was £5529. Inpatient physiotherapy accounted for a mean cost of £855 per patient, equivalent to 16.13 h of inpatient physiotherapy per patient. The smallest component of the cost determined by this Direct Inpatient Costing formula was theatre kit, which cost an estimated £309 per patient (Fig. 2).

**Table 1:** Cost breakdown by direct inpatient costing component

Cost component	Cost (£)
Theatre time	7952
Orthopaedic ward	5985
Surgical consumables and implants	5529
Critical care ward	1743
Inpatient physiotherapy	855
Theatre kit	309
Total (mean)	22,373

### Comparative analysis

**Fig. 3:**
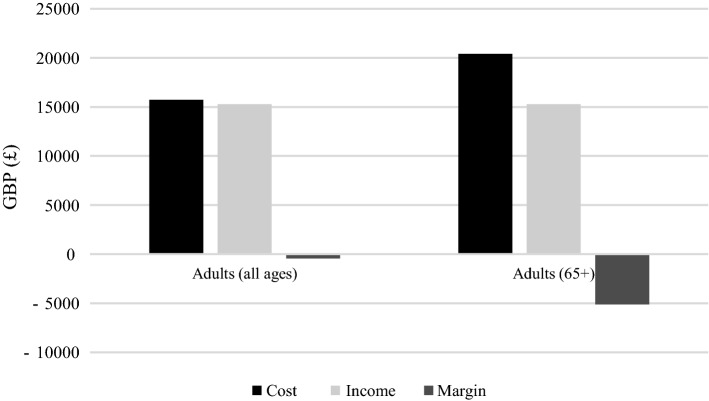
Calculated cost, income and margin for the older adults cohort in this study compared to findings from a previous study. *Source* Adapted from Tissingh et al. (2017) (Injury)

The median direct inpatient cost, income from HRG tariffs, and median margin for patients over the age of 65 years were compared to findings from a previous study with a similar methodology, which investigated the direct inpatient cost of treating open lower limb fractures in a general patient cohort [8]. The results from the two studies are demonstrated in Fig. 3. For older adults (65 years and over), the median direct inpatient cost was substantially higher than the median cost for the general cohort [8], resulting in a larger median margin (£5113 compared to £440), and thus loss to the hospital, for each patient over 65 years old compared to the general cohort. Direct inpatient costing analysis necessarily produces an underestimate of the true cost, which is likely higher by several thousand pounds [8]. The calculated costs from the younger patient cohort are used as published [8] and have not been adjusted for inflation, meaning that the difference in costs and margins between the two cohorts would be marginally smaller if inflation is taken into account. This is further addressed in the “Discussion” section.

**Fig. 4:**
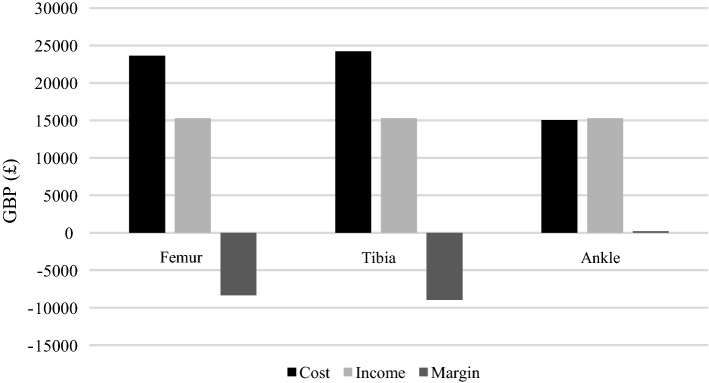
Calculated cost, income and margin by site of fracture, adults over 65

The cost of treatment within our study was also compared by the site of the open lower-limb fracture. Figure 4 displays the differences in median cost, and thus margin, between patients with a femur, tibia or ankle fracture. In this patient cohort, open fractures of the femur (£23,949) and tibia (£24,549) were more expensive to treat than fractures of the ankle (£15,362), with the consequence that femur and tibia fractures caused a significant loss for the hospital, at £8664 and £9264 per patient, respectively, while ankle fractures had only a small negative margin of £77 per patient (Table 2). A one-tailed unpaired *T*-test yielded a significant difference (*p* = 0.0007) between the cost of tibia and ankle fractures. The difference in cost between femur and ankle fractures was insignificant (*p* = 0.55), theorised to be due to the small sample size of open femur fractures.

**Table 2:** Median cost per fracture and resultant gains or losses

Patient group	Median cost (£)	Median margin (£)
Whole cohort	20,398	− 5113
Male	20,886	− 5601
Female	19,304	− 4019
Femur	23,949	− 8664
Tibia	24,549	− 9264
Ankle	15,362	− 77

## Discussion

There is a scarcity of primary literature and published clinical audits that have investigated the cost of orthopaedic treatment. This study used a direct inpatient costing formula to estimate the cost of treating 58 patients over the age of 65 years who were treated for an open lower limb fracture between March 2014 and March 2019 in a Major Trauma Centre. The median cost of treating one case, comprising theatre time, implants, inpatient physiotherapy and ward and critical care bed-nights, was £20,398 (mean £22,373). The median cost of treatment was compared to the average income received by the hospital for these injuries to determine the margin. Our centre suffered an average financial loss of £5113 per patient over 65 years treated for an open lower limb fracture. An average negative margin of £5113 per older patient with an open lower limb fracture in our centre is considerable. This amount of money in the NHS could be used to cover other important deficits, such as the salaries of additional nursing staff and doctors [21].

The income received by a Trust for the care it has provided is based on HRG cost codes, which are in turn constructed through clinical coding of the cases treated by the hospital(s). Inaccurate clinical coding has been shown to lead to huge losses for NHS hospitals [8, 22] and for the past 5 years, approximately 50% of Trusts have been in deficit [23]. Therefore, for hospitals to be appropriately remunerated for the treatments they provide, it is essential that the HRG tariffs are an authentic reflection of the true cost of care. The tariffs currently in place for the care of open lower limb fractures have not been updated since 2013–2014 and consequently do not reflect the rising costs of health care, including but not limited to inflation, novel treatments, new and significantly more expensive implants, as well as restructuring of treatment provision in the NHS. The average tariff for an open fracture is £8361 [8], a figure significantly lower than the cost calculated by this study and the limited available related literature.

This study found that the elderly patient cohort required significantly longer hospital stays, both on the orthopaedic ward and in critical care, than a previous study with a younger cohort. The patients over 65 years stayed on average 35.47 nights on the ward and 1.94 nights in critical care, compared to 17.73 and 1.32 nights, respectively, in the younger adult cohort [8]. The major trauma patient population in the UK is becoming more elderly, different to the archetypal young male trauma patient, and this is beginning to be recognised within the NHS [24]. Low impact trauma such as a fall from standing which would cause minor bruising to a healthy 20–40 year old leads to major injuries to frail tissues [24]. Approximately 30% of over 65s in Europe fall each year [25]. Falls risks include sensory impairment such as vision, polypharmacy, impaired mobility, comorbidities, cognitive impairment, nutritional deficiencies—all of which are more common in the elderly. Comorbidities play a large role in the mechanism of injury. Osteoporosis (through a variety of mechanisms including steroid use, menopause, bisphosphonates and increased alcohol use) is the most significant factor. Weak brittle bones require less impact to fracture and more easily pierce skin due to reduced dermal elasticity and atrophied muscles [26]. Recovery is also slower in the elderly, which contributes greatly to the cost. Comorbidities such as diabetes and COPD make operations with general anaesthesia more difficult, lead to higher rates of complications and hospital-acquired infections, and increase the likelihood of the patient requiring intensive care. Additionally, social factors play a prodigious role in the length of hospital stay and ongoing care requirements. Elderly trauma patients are more likely to require an intermediate stay in a rehabilitation hospital or social care at home following discharge [27]. This step-down care comes with a large surplus cost burden. Over 65s are likely to be retired, but enjoy high purchasing power and many contribute greatly to the volunteering sector of the economy, including childcare responsibilities. There are undoubtedly indirect costs from reduced economic participation that are easy to overlook. The term ‘silver trauma’ encapsulates the necessary differences in the care of elderly trauma patients to optimise their treatment. The Trauma and Audit Research Network’s (TARN) most recent report on major trauma in older people highlights deficiencies in the care of these elderly trauma patients, including most notably reduced trauma team activations and fewer senior reviews [28]. The Midlands Silver Trauma Group have proposed the trial of a Silver Safety Net to improve the patient pathway with early recognition and appropriate triaging of low impact falls with the potential to cause major injuries [29].

It is interesting to discuss the results of this study in the context of the most pertinent existing published study, which used direct inpatient and patient-level costing approaches to estimate the cost of treating open lower limb fractures in the National Health Service in a patient cohort of all ages [8]. What our study adds to this existing understanding is a differentiation of direct inpatient cost for this injury, not just for a cohort of older adults (65+), but also by sex and site of fracture. Tissingh et al. calculated a direct inpatient cost of £15,725 in their patient cohort (mean age 40 years), considerably lower than the cost of £20,398 calculated for older adults in this study (mean age 72.7 years), using an adapted but similar methodology. Comparing our findings with the previously published findings, the actual difference in cost between the cohorts would be slightly smaller if the published figures were retrospectively adjusted for inflation. Using Bank of England inflation figures for 2015–2019, the margin for the younger patient cohort would be £491 when adjusted for inflation [30], compared to £440 from the published data and £5113 for the older adults cohort in this study. Given the substantial difference in the cost of treating older patients with the same injury, it may be reasonable to differentiate clinical cost codes not only by injury severity but also by patient demographics, such as age, although more evidence will be required to fully evaluate this question. Compared to the younger patient cohort [8], the breakdown of the total cost also differed. Stay on the orthopaedic ward and inpatient physiotherapy constituted considerably larger portions of the total cost in the older adults (65+), while theatre time (which comprised more than half of the cost in the younger patient cohort) and surgical consumables and implants were relatively smaller shares of the total cost as a result. However, although inpatient bed-nights were a significant absolute and relative contributor to the comparatively greater cost of care in older adults, these components are not driving the major difference between cost and income for this important group of patients. According to the latest National Tariff Payment System, each additional bed-night is reimbursed with £246 [5]. Although this figure is greater than the cost of £169 estimated by the Trust for the cost of a bed-night on the orthopaedic ward, it is significantly less than the cost of £899 for a bed-night in critical care. Further, although the Trust's bed-night costs do not include generic overheads, since stay in critical care was much shorter (mean 35.47 nights) than orthopaedic ward stay (mean 1.94 nights), it can be concluded that reimbursement for hospital bed-nights is appropriate. Therefore, the biggest causes of the major difference between cost and income to the hospital for this group of patients are the cost of implants and theatre time.

The costs for male (median £20,886, mean £23,303) and female (median £19,304, mean £21,550) patients in this study were not statistically different (*p* = 0.34). Nevertheless, further studies with more patients should be conducted to quantify a possible cost difference by sex, and investigate what factors may underlie such a difference. The median costs of treating open fractures of the femur, tibia (condyles and shaft) and ankle (medial and/or lateral malleolus, talus) were £23,949, £24,549 and £15,362, respectively. The reasons that may underlie the significant cost difference between treating open fractures of the ankle compared to the long bones of the femur and tibia are unclear and should also be investigated further. There were no identifiable demographic differences between these sub-groups in the study.

There exist several limitations in the data used for this study, as well as in the employed methodology. Although the data used in this study are primary patient data that were directly extracted from the main clinical database used by the hospital, it is single-centre only. As a result, this study was only able to include 58 patients that fit the inclusion criteria of this study and had received treatment for their injury within the 5 years preceding data extraction. There was a large range in the costs calculated for individual patients, spanning from £3338 to £72,665. In light of these findings, future studies in this area may benefit from stricter inclusion criteria than age of patient and site and type of fracture, including taking into account patient death before treatment completion, the incidence of rare complications and an unusual number of repeat operations. Furthermore, future studies should consider employing additional costing methodologies to provide more holistic estimates of the cost of care. Direct inpatient costing does not include the cost of medications, imaging, additional staffing, hospital overhead and treatment in other centres, nor the likely large hidden costs of outpatient follow-up, community physiotherapy and rehabilitation, community nursing and social care. More comprehensive inpatient costing analysis, such as patient-level costing, could not be conducted retrospectively due to the availability of information recorded in the hospital's data system. The components included for costing in this study represent the bulk of inpatient care costs [8], and the inclusion of additional components would have drastically reduced the number of patients that could be included in the study and thus affected its statistical power. Further studies could prospectively record and cost all individualised aspects of care throughout the inpatient journey of a patient meeting the case definition at presentation, using a patient-level costing approach with activity-based costing principles, to provide a more granular and comprehensive assessment of the cost of inpatient care.

## Conclusion

Open lower limb fractures are resource-intensive injuries, accounting for a substantial proportion of the workload and expenditure of orthopaedic trauma units. Health care providers are often unaware of the cost implications of treatment decisions, and cost-effectiveness generally does not factor in clinical decision-making. There is poor dissemination of information on the cost of treatment alternatives at the national and the trust level. This study provides a valuable estimate of the cost of treating open lower limb fractures in patients over the age of 65 years in a Major Trauma Centre in England. The median cost of care calculated in this study was approximately £20,400 per patient, resulting in a financial loss to the hospital of approximately £5100 per patient. This study highlights the large losses incurred by hospitals in treating these cases, and supports revision of the remuneration structures in the National Health Service to adequately cover their cost. In particular, this study supports that cost codes should be differentiated with respect to patient age in addition to injury severity, given the evidence presented here that these fractures are significantly more expensive in older patients.
